# Electroacupuncture Pretreatment Mitigates Myocardial Ischemia/Reperfusion Injury via XBP1/GRP78/Akt Pathway

**DOI:** 10.3389/fcvm.2021.629547

**Published:** 2021-06-14

**Authors:** Nisha Wang, Jipeng Ma, Yan Ma, Linhe Lu, Chao Ma, Pei Qin, Erhe Gao, Mingzhang Zuo, Jian Yang, Lifang Yang

**Affiliations:** ^1^Department of Anesthesiology, Xi'an Children's Hospital, Xi'an Jiaotong University, Xi'an, China; ^2^Department of Cardiovascular Surgery, Xijing Hospital, Air Force Medical University, Xi'an, China; ^3^Department of Anaesthesiology, Beijing Hospital, National Center of Gerontology, Institute of Geriatric Medicine, Chinese Academy of Medical Science, Beijing, China; ^4^Center for Translational Medicine, Lewis Katz School of Medicine at Temple University, Philadelphia, PA, United States

**Keywords:** electroacupuncture, myocardial ischemia/reperfusion injury, XBP1s, apoptosis, Akt

## Abstract

Myocardial ischemia/reperfusion injury is a common clinical problem and can result in severe cardiac dysfunction. Previous studies have demonstrated the protection of electroacupuncture against myocardial ischemia/reperfusion injury. However, the role of X-box binding protein I (XBP1) signaling pathway in the protection of electroacupuncture was still elusive. Thus, we designed this study and demonstrated that electroacupuncture significantly improved cardiac function during myocardial ischemia/reperfusion injury and reduced cardiac infarct size. Electroacupuncture treatment further inhibited cardiac injury manifested by the decrease of the activities of serum lactate dehydrogenase and creatine kinase-MB. The results also revealed that electroacupuncture elevated the expressions of XBP1, glucose-regulated protein 78 (GRP78), Akt, and Bcl-2 and decreased the Bax and cleaved Caspase 3 expressions. By using the inhibitor of XBP1 *in vitro*, the results revealed that suppression of XBP1 expression could markedly increase the activities of lactate dehydrogenase and creatine kinase-MB and cell apoptosis, thus exacerbating stimulated ischemia/reperfusion-induced H9c2 cell injury. Compared with stimulated ischemia/reperfusion group, inhibition of XBP1 inhibited the downstream GRP78 and Akt expressions during stimulated ischemia/reperfusion injury. Collectively, our data demonstrated that electroacupuncture treatment activated XBP1/GRP78/Akt signaling to protect hearts from myocardial ischemia/reperfusion injury. These findings revealed the underlying mechanisms of electroacupuncture protection against myocardial ischemia/reperfusion injury and may provide novel therapeutic targets for the clinical treatment of myocardial ischemia/reperfusion injury.

## Introduction

Ischemic heart disease is a common cardiovascular problem with high morbidity and mortality (1). Although it is important to timely restore the blood flow of an ischemic myocardium, cardiovascular outcomes can be further aggravated by the so-called myocardial ischemia/reperfusion (MI/R) injury. Therefore, exploring a safe and effective treatment is urgently needed to mitigate MI/R-induced injury.

The endoplasmic reticulum (ER) is an organelle where the target proteins are processed so as to prompt its post-translational modifications, proper folding, and protein transport (2). However, under cellular stress, the imbalance of ER protein processing and accumulation of the unfolded proteins and/or misfolded proteins results in ER stress, which further induces the unfolded protein response (UPR) as an adaptive response to restore ER homeostasis (3, 4). The UPR is initiated by three classical ER transmembrane sensors: protein kinase R-like ER kinase (PERK), inositol-requiring kinase 1 (IRE1), and activating transcription factor 6 (ATF6) (3). As a key player of ER stress, X-box binding protein I (XBP1) expression was induced by ATF6 and then was spliced by IRE1 (5). The resulting spliced form of XBP1 can further activate UPR to cope with ER stress (5). Previous studies further revealed that ER stress participated in the pathogenesis of numerous cardiac diseases (6). In transverse aortic constriction (TAC)-induced hypertrophic and falling hearts, sustained ER stress resulted in cardiomyocyte apoptosis and contributed to the progression from cardiac hypertrophy to heart failure through the canonical and non-canonical pathways (7, 8). Surprisingly, hypoxia induced ATF6 and glucose-regulated protein 78 (GRP78) expressions and protected cardiomyocytes from ischemic injury (9). ATF6 transgenic mice alleviated MI/R damage via decreasing oxidative stress, enhancing catalase expression, and reducing cell necrosis and apoptosis (10, 11). Notably, GRP78 as an XBP1 target was shown to stimulate Akt pathway to protect hearts from I/R injury (12). Furthermore, XBP1 can protect β-cells from lipotoxicity via activation of Akt pathway (13). However, the role of XBP1–Akt pathway in the MI/R injury was not fully investigated.

Based on the Chinese traditional medical theory, acupuncture is used to treat diseases for more than 2000 years (14, 15). Especially, electroacupuncture (EA) pretreatment at specific acupoints has been demonstrated as an effective approach to improve cardiac function in diverse pathological conditions. In spontaneously hypertensive rats, long-term EA reduced the wall thickness of left ventricle via regulating the NOS pathway (16). It was revealed that EA at PC6 (Neiguan) and PC5 (Jianshi) acupoints reduced myocardial malondialdehyde (MDA) level, norepinephrine concentration, and cell apoptosis to attenuate MI/R injury in animal models (17, 18). Furthermore, in our previous clinical study, EA pretreatment significantly attenuated MI/R injury in patients with heart valve replacement surgery (19). However, whether EA pretreatment regulates ER stress signaling pathways to protect hearts against MI/R injury has not been explored previously.

Thus, the present study was designed to investigate the protection of EA pretreatment against MI/R injury and the potential role of XBP1/GRP78 signaling in this process. These findings may provide the theoretical basis for the clinical use of EA pretreatment against MI/R injury.

## Materials and Methods

### Animal Model of Myocardial Ischemia/Reperfusion Injury

The animal experimental protocol in this study was approved by the Animal Care Committee of Air Force Medical University. All animal procedures were performed in accordance with the Guidelines for the Care and Use of Laboratory Animals by the Institute of Laboratory Animal Research from US National Institutes of Health (National Institutes of Health Publication No. 8523, revised 1996). Male C57BL/6 mice aged 10~12 weeks and weighing 22~26 g were obtained from the Laboratory Animal Center of Air Force Medical University. All mice were housed at 20–25°C under a 12-h light/dark cycle and received a standard diet and water *ad libitum*.

MI/R injury mouse model was established according to the previous study (20). Briefly, mice were anesthetized with 1–2% isoflurane via an isoflurane vaporizer (Matrx, Orchard Park, NY, USA). A skin scar was cut in the left chest, and a tiny hole was made at the fourth intercostal space. Afterwards, the heart was smoothly squeezed out of the thoracic cavity. The left anterior descending (LAD) coronary artery was ligated by a 6-0 silk suture for 30 min, and then the slipknot was released. The reperfusion phase lasted for 2–4 h, and the heart samples were collected for protein expressions analysis. The cardiac function, myocardial infarct size, cell apoptosis, and lactate dehydrogenase (LDH) as well as creatine kinase-MB (CK-MB) were assessed following a 24-h reperfusion. The same procedure except ligation of LAD was performed in the mice of sham group.

### Electroacupuncture Pretreatment

EA pretreatment was conducted by using the Hwato Electronic Acupuncture Treatment Instrument (Suzhou Medical Appliances, Suzhou, China). Briefly, mice were anesthetized and maintained by inhalation of 1–2% isoflurane via an isoflurane vaporizer. The needles connected to the electrodes were inserted into 2- to 3-mm depth of muscle layers at the Neiguan acupoint (PC6) of both forelimbs, which are located between the palmar tendon and flexor carpi ulnaris (21). Mice were stimulated at the density of 1 mA with a frequency of 2/15 Hz for 30 min once a day for 3 days. The MI/R surgery or sham surgery was performed within the 30 min after the last EA treatment. Mice from Sham and Sham+EA groups were anesthetized for 30 min to avoid the effects of isoflurane between different groups.

The mice were divided into the following four groups with 12–15 mice for each: sham group (Sham), sham group with EA pretreatment (Sham+EA), MI/R injury group (MI/R), and MI/R group with EA pretreatment (MI/R+EA). Mice in the Sham+EA or MI/R+EA groups received EA preconditioning for 3 consecutive days followed by sham or MI/R surgery, while mice in the Sham or MI/R group underwent the sham or MI/R surgery, respectively.

### Echocardiography

Mice were anesthetized and maintained by inhalation of 1–2% isoflurane after 24-h reperfusion. Cardiac function was evaluated by Doppler echocardiography with a 15-MHz linear transducer (Visual Sonic Vevo 2100, Toronto, ON, Canada). Mice were placed on a heating pad to maintain the body temperature during the whole procedure. M-mode echocardiography was recorded and used to assess cardiac function. Left ventricular ejection fraction (LVEF) and left ventricular fractional shortening (LVFS) were obtained by using Vevo LAB 3.1.0 software.

### Activities Measurements of Lactate Dehydrogenase and Creatine Kinase-MB

The serum was obtained by centrifugation of mouse blood at 3,000 rpm for 10 min after 24-h reperfusion and used for LDH and CK-MB determination. The assay was conducted according to the manufacturer's instruction (Jiancheng Bioengineering, Nanjing, China). The activities of LDH and CK-MB were calculated based on the methods described in the manufacturer's instruction.

### Triphenyltetrazolium Chloride/Evans Blue Double Staining

The mice were anesthetized with 1–2% isoflurane, and the LAD was occluded at the same position following 24-h reperfusion. The 3% Evans blue solution was injected into the hearts via the aorta to stain the non-ischemic area of the heart. The whole hearts were then collected and frozen on dry ice for 10 min and cut into four slices transversally from the bottom of the hearts. The slices were stained in 1.5% triphenyltetrazolium chloride (TTC) in phosphate solution (pH 7.4) and incubated at 37°C for 20 min. Then the slices were fixed in 4% paraformaldehyde for 12 h, and the images were obtained by using a digital camera. The infarct size was calculated by the ratio of white area to white and red areas. The size was determined by using Image-Pro Plus software (Media Cybernetics, Inc., Rockville, MD, USA).

### Terminal Deoxynucleotidyl Transferase-Mediated Dutp Nick-End Labeling Staining

Myocardial apoptosis and cell apoptosis were determined by an *in situ* cell death detection kit (Roche Molecular Biochemicals, Mannheim, Germany) as previously described (22). In brief, at the end of the experiment, the myocardial tissues and H9c2 cells were fixed in 4% paraformaldehyde for at least 24 h. After the paraffin-imbedded sections were prepared, the manufacturer's instruction for TUNEL staining was followed. The apoptotic myocardial cells and H9c2 cells were stained with TUNEL staining solution, and nuclei were visualized by DAPI staining. Then the images were obtained with an Olympus FV10i microscope (Olympus, Tokyo, Japan); and an apoptotic rate was presented as the count of TUNEL-positive cardiomyocytes to the total number of cells.

### Cell Culture and Stimulated Ischemia/Reperfusion Model

H9c2 cells were cultured in Dulbecco's Modified Eagle Medium (DMEM; HyClone, Logan, UT, USA) with 10% fetal bovine serum (Gibco, Grand Island, NY, USA) at 37°C in a 5% CO_2_ air incubator. The experiments included four groups: (1) the cells in the CON group were cultured in the serum-free DMEM. (2) H9c2 cells in stimulated I/R (SI/R) group were cultured in serum-free DMEM for 12 h and then subjected to the ischemic buffer (10 mM of deoxyglucose, 137 mM of NaCl, 12 mM of KCl, 0.49 mM of MgCl_2_, 0.9 mM of CaCl_2_·2H_2_O, 0.75 mM of sodium dithionate, 20 mM of lactate, and 4 mM of Hepes, pH 6.5) for 2 h. The cells were then cultured in normal DMEM at 37°C in an incubator (5% CO_2_, 95% air) for 4 h to establish *in vitro* SI/R model. (3) The cells in XI group (XBP1 inhibitor, 4μ8C) were cultured in the serum-free DMEM and then incubated with 5 μM of 4μ8C for 2 h. (4) The cells in SI/R+XI group were incubated 5 μM of 4μ8C for 2 h and then subjected to ischemic medium for 2 h. Then the cells were cultured in normal DMEM at 37°C in an incubator (5% CO_2_, 95% air) for 4 h. The supernatant of cell culture was collected for LDH and CK-MB activities measurements following the protocol described above.

### Western Blotting

The proteins were isolated from the heart left ventricles including the infarct zone and broader zone and H9c2 cells for western blotting detection. The proteins were separated with sodium dodecyl sulfate–polyacrylamide gel electrophoresis (SDS-PAGE) and then transferred on to a polyvinylidene difluoride (PVDF) membrane (Millipore, Billerica, MA, USA); subsequently, the membrane is incubated with 5% non-fat milk in TBST. Western blotting was then performed with antibodies against XBP1 (Cat. 83418, 1:1,000; Cell Signaling Technology, Danvers, MA, USA), GRP78 (Cat. 3183, 1:1,000; Cell Signaling Technology), p-Akt (Cat. 4060, 1:1,000; Cell Signaling Technology), Akt (Cat. 4691, 1:1,000; Cell Signaling Technology), Bcl-2 (Cat. ab196495, 1:1,000; Abcam, Cambridge, UK), Bax (Cat. 2772, 1:1,000; Cell Signaling Technology), cleaved Caspase 3 (Cat. 9664, 1:1,000; Cell Signaling Technology), and GAPDH (Cat. AT0002, CMC TAG, 1:5,000). After that, the proteins were probed with horseradish peroxidase (HRP)-conjugated secondary antibodies and visualized by a ChemiDoc Imaging System (Bio-Rad Laboratories, Hercules, CA, USA). Then the relative quantification of proteins was presented as the ratio of target proteins to GAPDH.

### Statistical Analysis

All data were presented as mean ± SD. Data were analyzed with the GraphPad Prism Software version 7.0 (GraphPad Software, San Diego, CA, USA). Normality analysis of data was performed by the Shapiro–Wilk test. Statistical significance (*P* < 0.05) was estimated by one-way ANOVA followed by Bonferroni correction for *post-hoc t*-test.

## Results

### Electroacupuncture Pretreatment Protected Cardiac Function Against Myocardial Ischemia/Reperfusion Injury

**Figure 1:**
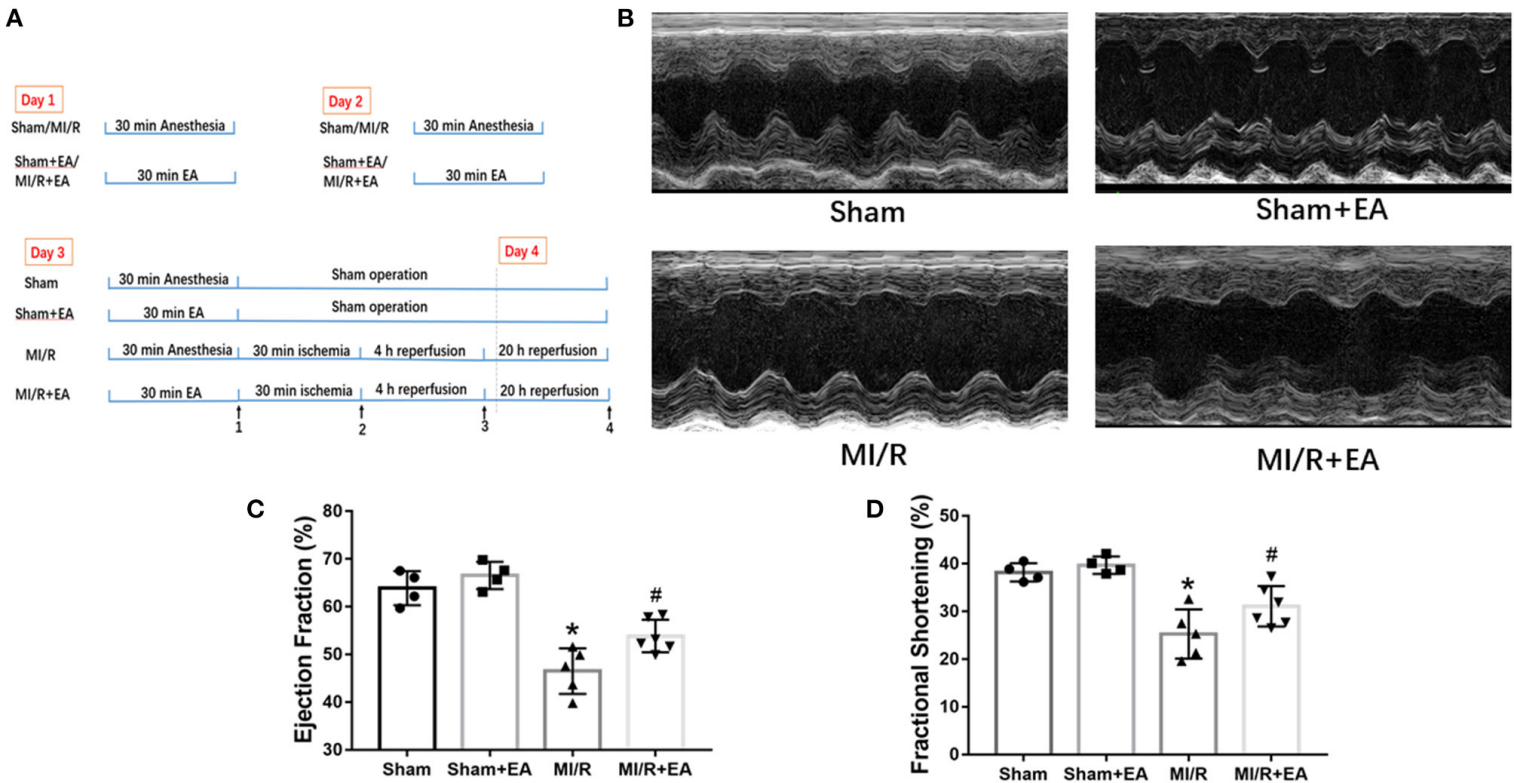
EA pretreatment mitigated MI/R-induced cardiac dysfunction. **(A)** A schematic illustration of the study groups and *in vivo* experimental protocols. Time point 1: ischemic phase. Time point 2: reperfusion phase. Time point 3: heart tissue collection for protein analysis. Time point 4: echo analysis and TTC staining. **(B)** Representative images of M-mode echocardiography in MI/R-induced cardiac injury following EA pretreatment. Images of M-mode echocardiography were recorded from horizontal direction of the heart and then were analyzed to assess cardiac systolic function. **(C)** Ejection fraction. **(D)** Fractional shortening. *n* = 4–6 in each group. EA, electroacupuncture; MI/R, myocardial ischemia/reperfusion; TTC, triphenyltetrazolium chloride. *P < 0.05 vs. Sham group, ^#^*P* < 0.05 vs. MI/R group.

To evaluate the effects of EA on cardiac function following MI/R injury and EA treatment, echocardiography was performed. M-mode images were obtained to measure LVEF and LVFS so as to evaluate cardiac contractile function. As shown in Figure 1, MI/R significantly reduced LVEF and LVFS compared with sham group, while EA pretreatment for 3 consecutive days greatly increased LVEF and LVFS compared with MI/R group. These data demonstrated that MI/R resulted in compromised cardiac function, while EA pretreatment improved cardiac function, which was impaired by MI/R injury. However, cardiac function in EA+sham group was not significantly altered compared with sham group, implying no obvious effects on cardiac function by EA pretreatment in sham-operated mice. These results data together revealed that EA pretreatment elevated MI/R-induced reduction of LVEF and LVFS, thus protecting hearts from MI/R injury.

### Electroacupuncture Pretreatment Reduced Myocardial Infarct Size and the Activities of Lactate Dehydrogenase and Creatine Kinase-MB

**Figure 2:**
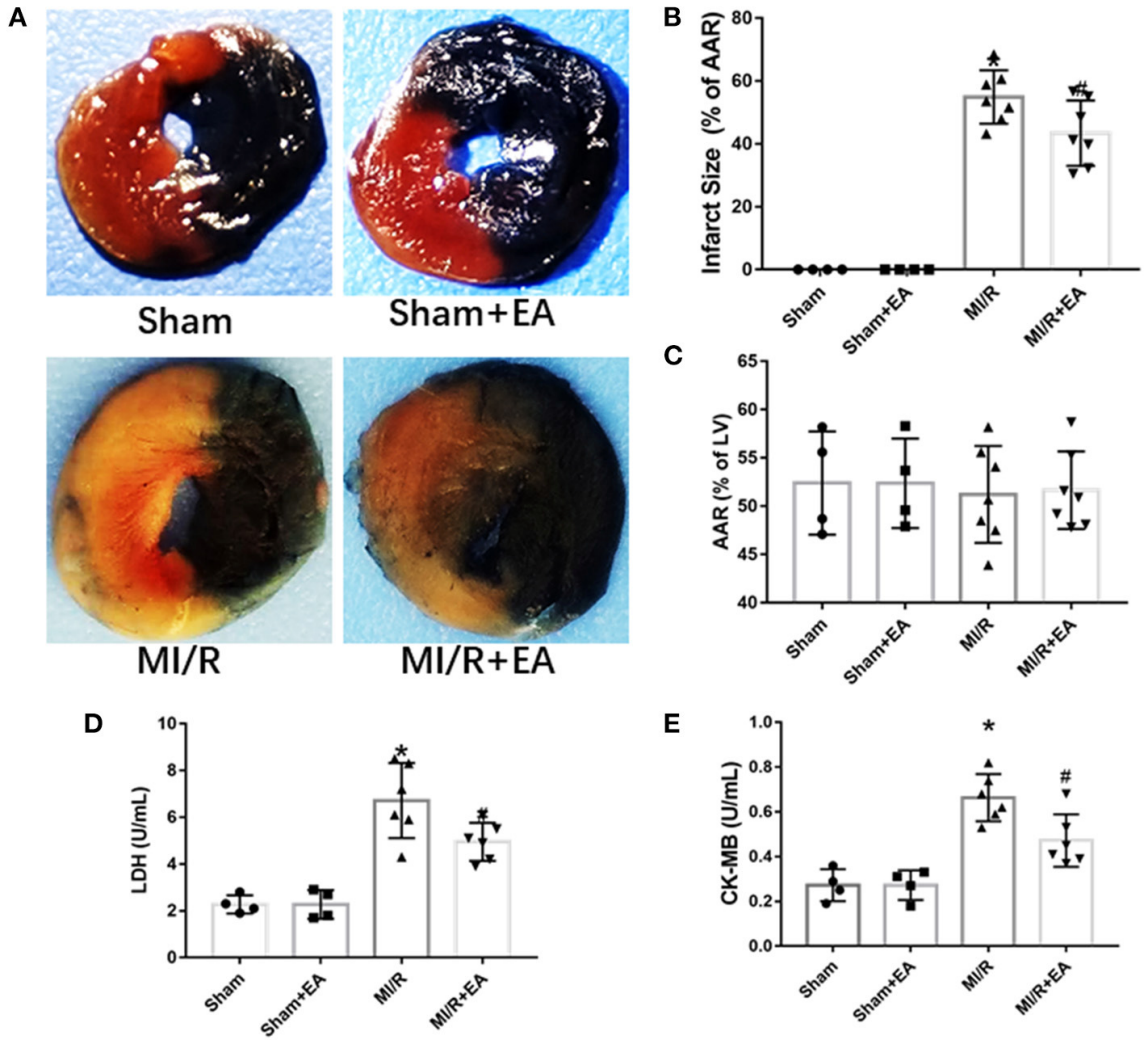
The effects of EA pretreatment on myocardial infarct size, LDH, and CK-MB activities in MI/R-induced cardiac injury. **(A)** Representative images of cardiac slices by TTC/Evans blue double staining in MI/R-induced cardiac injury following EA pretreatment. **(B)** Myocardial infarct size of different groups. **(C)** Myocardial area at risk (AAR) following MI/R procedure and EA treatment. *n* = 4–7 in each group for TTC/Evans blue double staining. **(D)** Serum LDH activity. **(E)** Serum CK-MB activity. *n* = 4–6 in each group for enzyme activity measurements. LDH, lactate dehydrogenase; CK-MB, creatine kinase-MB isoform; EA, electroacupuncture; MI/R, myocardial ischemia/reperfusion; TTC, triphenyltetrazolium chloride. *P < 0.05 vs. Sham group, ^#^*P* < 0.05 vs. MI/R group.

To further investigate whether EA pretreatment could affect MI/R-induced myocardial infarct size and myocardial cell death, TTC/Evans blue double staining was used to assess infarct size; and LDH and CK-MB activities were determined to evaluate myocardial cell death. Our results showed that MI/R led to a significant increase of myocardial infarct size compared with sham group, while EA pretreatment dramatically reduced myocardial infarct size compared with MI/R group as shown in Figure 2. The above result clearly showed that EA pretreatment significantly reduced MI/R-induced myocardial infarct size. Furthermore, the activities of LDH and CK-MB in the serum were increased in response to MI/R injury compared with sham group, while EA pretreatment decreased the activities of LDH and CK-MB in EA+MI/R group compared with MI/R group as shown in Figure 2. These data revealed that myocardial cell death caused by MI/R injury was inhibited following EA pretreatment. Taken together, these results indicated that EA pretreatment decreased myocardial infarct size and LDH and CK-MB release to protect hearts from MI/R injury.

### Electroacupuncture Reduced Myocardial Ischemia/Reperfusion-Induced Cell Apoptosis and Activated XBP1/GRP78/Akt Pathway

**Figure 3:**
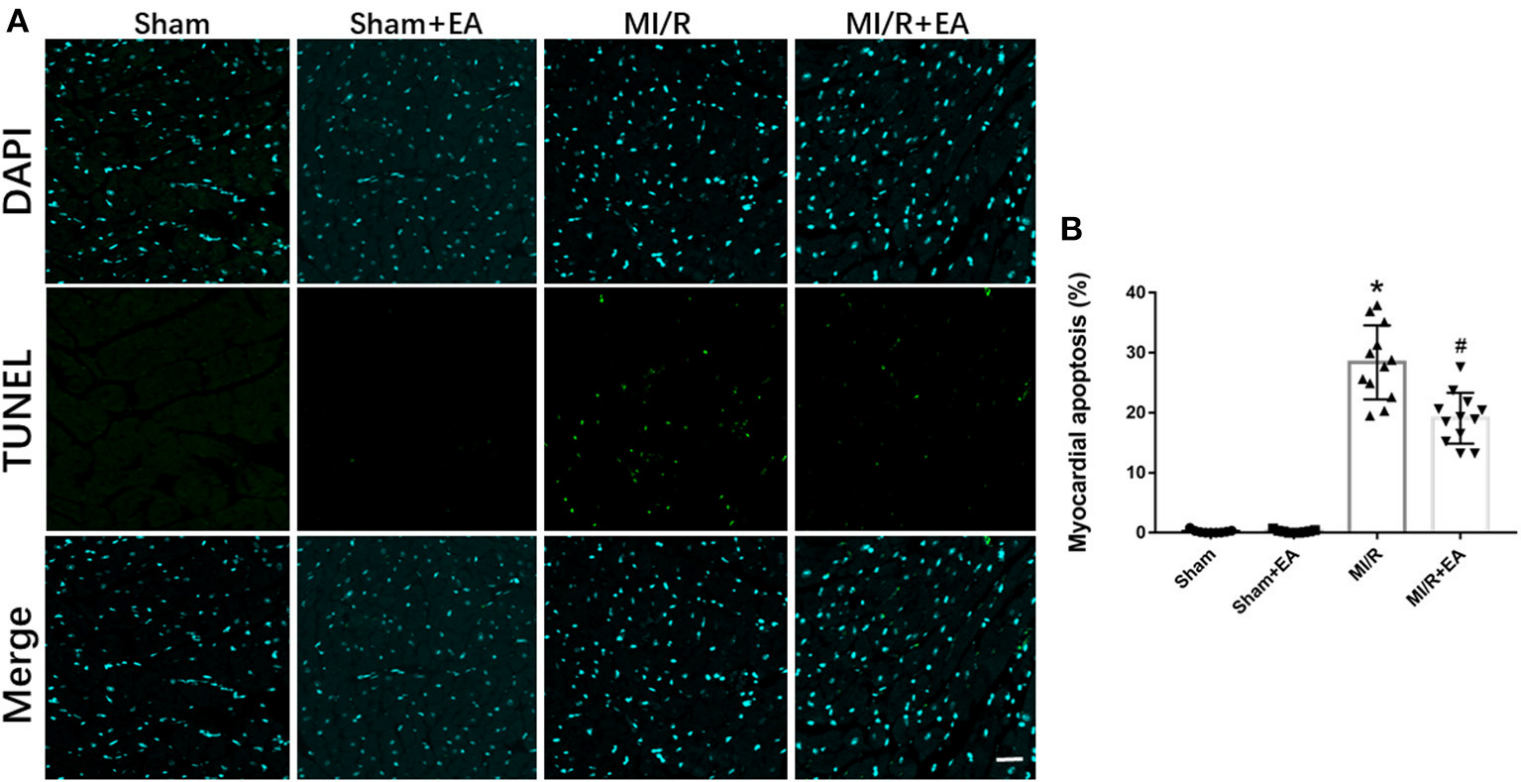
The effects of EA pretreatment on myocardial apoptosis in MI/R-induced cardiac injury. **(A)** Representative images of myocardial apoptosis by TUNEL staining in MI/R-induced cardiac injury following EA pretreatment. **(B)** Myocardial apoptosis of mice from different groups. *n* = 3–4 for each group. Three to four hearts in each group were used for TUNEL staining, and five random fields in three sections of each heart were used for further analysis. Bar, 100 μm. *P < 0.05 vs. Sham group, ^#^*P* < 0.05 vs. MI/R group. EA, electroacupuncture; MI/R, myocardial ischemia/reperfusion.

To elucidate the role of cell apoptosis in EA protection against MI/R injury, cell apoptosis among different groups was determined. As shown in Figure 3, MI/R significantly enhanced myocardial apoptosis compared with sham group. Moreover, EA pretreatment decreased MI/R-induced cell apoptosis by 27.6% compared with MI/R group, implying the protective role of EA on reducing cell apoptosis during MI/R injury.

**Figure 4:**
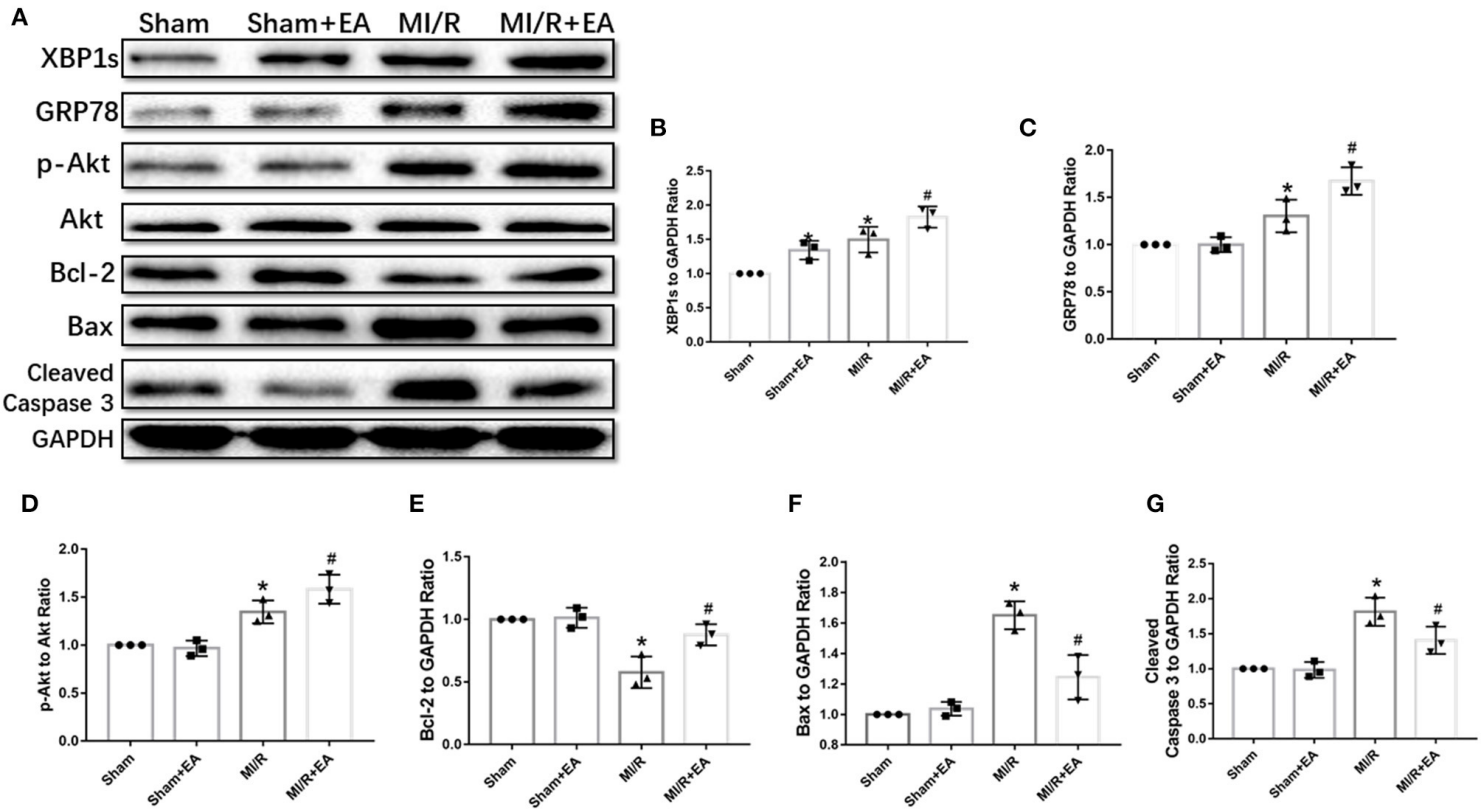
EA pretreatment activated XBP1/GRP78/Akt pathway and reduced apoptotic protein expressions. **(A)** Representative blot images of proteins related to XBP1/GRP78/Akt and apoptotic signaling pathways. **(B–G)** The quantitative analysis of XBP1, GRP78, p-Akt, Bcl-2, Bax, and cleaved Caspase 3 protein expressions. *n* = 3 in each group. *P < 0.05 vs. Sham group, ^#^*P* < 0.05 vs. MI/R group. EA, electroacupuncture; MI/R, myocardial ischemia/reperfusion.

To further investigate the potential molecular mechanisms underlying EA protection against MI/R injury, the expressions of XBP1/GRP78/Akt signaling pathway and apoptotic proteins were assessed. As shown in Figure 4, our data demonstrated that MI/R injury resulted in increase of XBP1, GRP78, and phosphorylated Akt (p-Akt) expressions, while the pro-apoptotic proteins including Bax and cleaved Caspase 3 were upregulated compared with sham group. The results showed that the expressions of XBP1, GRP78, and Akt were further increased, and the expressions of Bax and cleaved Caspase 3 were decreased following EA pretreatment. These results indicated that the molecular mechanism of EA protection against MI/R injury was at least partly via activation of XBP1/GRP78/Akt signaling pathway, therefore ultimately reducing cell apoptosis.

### Inhibition of XBP1 Exacerbated Stimulated Ischemia/Reperfusion Injury and Cell Apoptosis in H9c2 Cells

**Figure 5:**
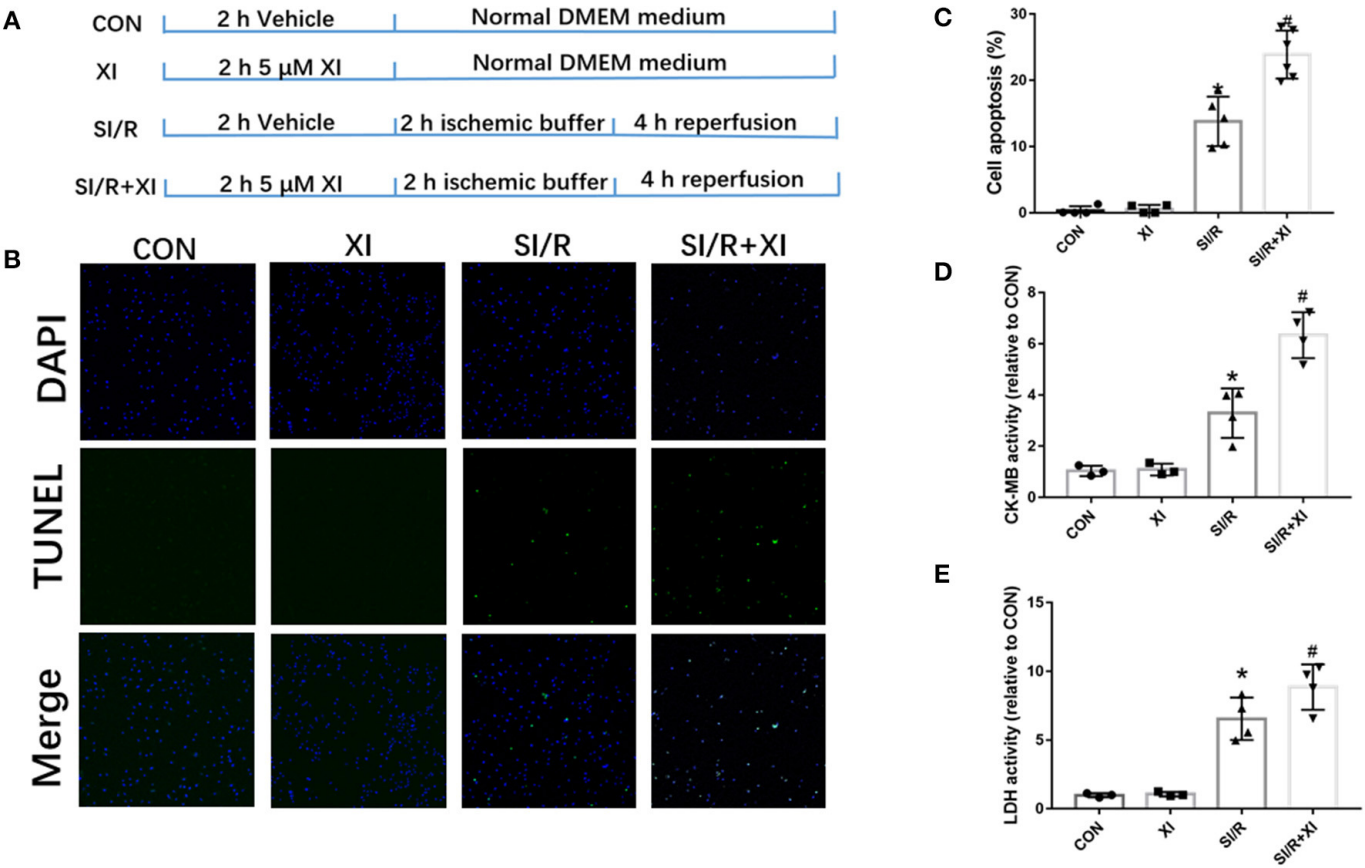
4μ8C, an XBP1 inhibitor, increased H9c2 cell apoptosis LDH and CK-MB activities *in vitro*. **(A)** A schematic illustration of the study groups and *in vitro* experimental protocols. **(B)** Representative images of cell apoptosis by TUNEL staining in SI/R-induced cell injury following 4μ8C pretreatment. **(C)** The quantitative analysis of cell apoptosis following 4μ8C pretreatment. Eight random fields were selected for quantitation of cell apoptosis for each repeat experiment. **(D)** LDH activity. **(E)** CK-MB activity. *n* = 3–4 in each group. XI, XBP1 inhibitor; LDH, lactate dehydrogenase; CK-MB, creatine kinase-MB; SI/R, stimulated ischemia/reperfusion. Bar, 50 μm. *P < 0.05 vs. CON group, ^#^*P* < 0.05 vs. SI/R group.

To further investigate the role of XBP1-mediated signaling pathway in SI/R-induced cell injury, the inhibitor 4μ8C was used in *in vitro* study. The results in Figure 5 revealed that XBP1 inhibitor 4μ8C significantly exacerbated SI/R-induced H9c2 cell injury manifested by the increase of LDH and CK-MB activities and the elevation of cell apoptosis compared with the SI/R group. However, 4μ8C alone did not significantly affect the activities of LDH and CK-MB or cell apoptosis in normal conditions. These results showed that XBP1 inhibition could deteriorate SI/R-induced cell injury via increase of cell death.

### XBP1 Inhibition Downregulated GRP78/Akt Pathway in Stimulated Ischemia/Reperfusion-Injured H9c2 Cells

**Figure 6:**
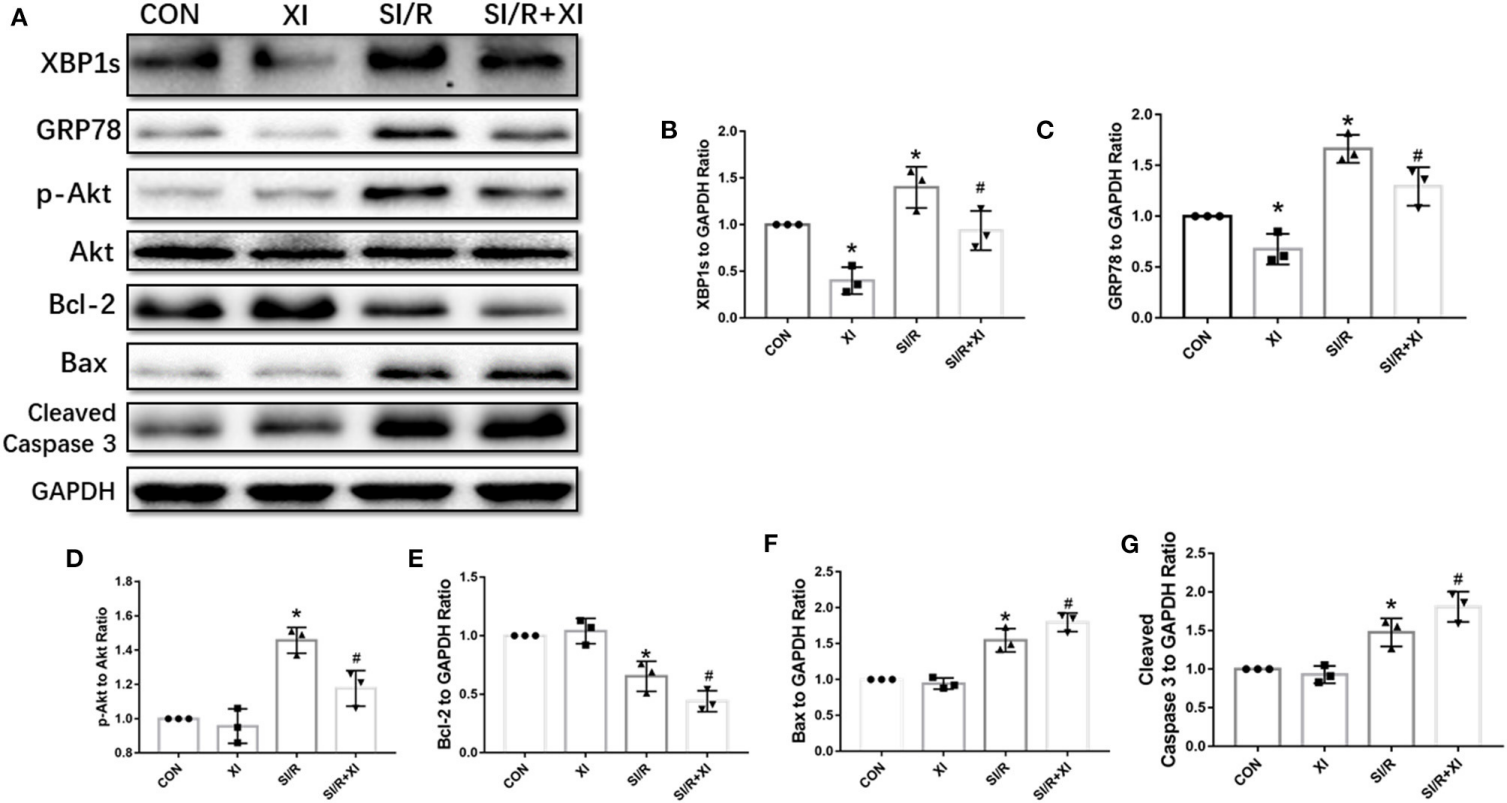
4μ8C inhibited XBP1/GRP78/Akt pathway and enhanced apoptotic protein expressions *in vitro*. **(A)** Representative blot images of proteins related to XBP1/GRP78/Akt and apoptotic signaling pathways in H9c2 cells. **(B–G)** The quantitative analysis of XBP1, GRP78, p-Akt, Bcl-2, Bax, and cleaved Caspase 3 protein expressions. *n* = 3 in each group. *P < 0.05 vs. CON group, ^#^*P* < 0.05 vs. SI/R group.

To demonstrate the effects of XBP1 inhibition on GRP78/Akt signaling pathway, we further explored the effects of 4μ8C on the XBP1/GRP78/Akt pathway. The western blotting in Figure 6 demonstrated that the XBP1 inhibitor 4μ8C downregulated XBP1, GRP78, Akt, and Bcl-2 expressions and elevated the expressions of Bax and cleaved Caspase 3 compared with MI/R group. Our *in vitro* results favored the notion that the inhibition of XBP1 could worsen SI/R-induced cell injury via regulation of GRP78/Akt pathway.

## Discussion

The present study demonstrated that EA pretreatment attenuated MI/R-induced cardiac dysfunction and mitigated MI/R-induced damage by decreasing serum LDH, CK-MB, and myocardial apoptosis. The underlying molecular mechanism of EA protection was shown to be involved in the activation of XBP1/GRP78/Akt pathway. Further *in vitro* result revealed that inhibition of XBP1 decreased the downstream GRP78 and Akt expressions and elevated cell apoptosis, implying the importance of XBP1-mediated pathway against MI/R injury.

As a traditional Chinese medical therapy, EA is shown to be a beneficial treatment for several diseases including stress urinary incontinence, knee osteoarthritis, and acute ischemic cerebral apoplexy in clinical studies (23–25). Notably, EA significantly reduced myocardial injury induced by cardiac hypertrophy and ischemic insult in animal models and clinical studies (18, 19). EA pretreatment at Neiguan (PC6) acupoint mitigated cardiac hypertrophy via upregulation of ERK signaling pathway (26). Furthermore, Lujan et al. demonstrated that EA decreased the incidence of I/R-mediated ventricular tachyarrhythmias via lowering cardiac metabolic demand (27). Inhibition of cardiac norepinephrine release and regulation of opioid and PKC-dependent pathways by EA treatment attenuated MI/R injury in a rabbit model (17). Consistent with these previous studies, we observed that EA pretreatment for 3 consecutive days reduced myocardial infarct size, increased LVEF and LVFS, but suppressed the activities of LDH and CK-MB in MI/R injury. Moreover, EA pretreatment decreased the levels of expressions of Bax and Cleaved Caspase 3. These results clearly showed that EA pretreatment attenuated MI/R-induced cardiac contractile dysfunction and myocardial apoptosis.

Accumulating evidence has demonstrated that ER stress was markedly activated in I/R-injured myocardium (28, 29). ER stress is known to occur when the protein synthesis and protein process exceed its capacity in ER lumen under cellular stress. The UPR can be initiated by three signaling pathways including eIF2a-ATF4, IRE1a-XBP1, and ATF6 pathways to maintain ER homeostasis (30). Moreover, melatonin inhibited PERK-eIF2α-ATF4-mediated ER stress to protect cardiac function from MI/R injury (31). It was also revealed that ischemic preconditioning attenuated MI/R-induced injury through suppression of ER stress (32). All these data favored the notion that the inhibition of ER stress would be beneficial to mitigate myocardial ischemic injury. However, these protective effects were not observed by direct inhibition of key molecules in ER stress but the upstream regulators of ER stress. In contrast to these results, acute activation of ER stress by the key molecules involved in ER stress signaling pathway displayed cardioprotective roles in ischemic heart diseases, which was even attributed to the other molecular mechanisms beyond ER stress signaling pathways. ATF6 as a key mediator of one conserved branch of ER stress protected hearts from MI/R injury via inducing the expressions of catalase and protein disulfide isomerase (10, 11). Furthermore, ATF6 binds to the promoter of the protein disulfide isomerase associated 6 (*pdia6*) gene to protect cardiomyocytes against simulated I/R-induced death *in vitro* (33). GRP78 as a signal sensor of ER stress activated by ischemic preconditioning attenuated ischemic injury in cardiomyocytes via activation of Nrf2/HO-1 pathway (34, 35). Cardiomyocyte-specific overexpression of GRP78 protected hearts from MI/R injury *in vivo* and *in vitro* through stimulation of Akt signaling pathway (12). Moreover, this study revealed that GRP78 could interact with phosphatidylinositol 3-kinase and therefore lead to the stimulation of Akt (12). Thus, our present study was designed to investigate whether activation of XBP1 could activate GRP78/Akt signaling pathway to protect hearts from MI/R injury. Our results revealed that EA pretreatment markedly upregulated XBP1 expression and the downstream GRP78 expression during MI/R injury. Then GRP78 further enhanced Akt signaling to reduce MI/R-induced cell apoptosis. Additionally, in an *in vitro* SI/R model, inhibition of XBP1 could significantly reduce cell viability and increase cell apoptosis and the activities of LDH and CK-MB, indicating a vital role of XBP1 in the pathology of MI/R injury.

The key role of Akt signaling in the pathogenesis of MI/R injury has been illustrated in numerous previous studies. As an important anti-apoptotic pathway, promotion of Akt signaling pathway significantly reduced cell apoptosis to protect hearts and cardiomyocytes from I/R injury (36–38). Our data consistently showed that cell apoptosis determined by TUNEL staining and apoptotic protein expressions was significantly reduced by the induction of XBP1/GRP78/Akt axis. The inhibition of XBP1 in H9c2 cells inhibited Akt phosphorylation, thus execrating cell injury and apoptosis. Our study clearly showed that the inhibition of XBP1 worsened SI/R-induced cell injury.

However, there are some limitations in the present study. First, although we demonstrated that EA pretreatment could increase XBP1 expression, the mechanisms and the upstream regulators of XBP1 were still absent and need further exploration. We speculate that EA pretreatment may lead to the overall metabolic changes of the muscle and alter the profile of myokines. Second, the time point of EA treatment was before MI/R surgery. This will limit the clinical use, and the post-ischemia treatment is more reasonable for clinical application. Third, the *in vitro* data can only demonstrate that the XBP1/GRP78/Akt pathway participates in SI/R-induced cell injury since EA cannot directly treat cell *in vitro*.

In summary, the results of this study suggest for the first time that EA pretreatment upregulated XBP1/GRP78/Akt signaling pathway and improved cardiac function during MI/R injury. Clarification of upstream of XBP1 in the pathological process of MI/R injury will be required to better understand the action of EA protection, which may not only contribute to elucidation of the molecular mechanism but have potential clinical use.

## Data Availability Statement

The raw data supporting the conclusions of this article will be made available by the authors, without undue reservation.

## Ethics Statement

The animal study was reviewed and approved by the Animal Care Committee of Air Force Medical University.

## Author Contributions

LY, JY, and MZ designed and supervised the study and revised the manuscript. NW, JM, YM, and CM conducted the experiments and collected the data. LL and PQ collected and analyzed the data. EG established the animal model. NW and JM wrote the draft. All authors approved the final manuscript.

## Conflict of Interest

The authors declare that the research was conducted in the absence of any commercial or financial relationships that could be construed as a potential conflict of interest.
